# Delayed fixation of displaced type II and III pediatric femoral neck fractures

**DOI:** 10.4103/0019-5413.53455

**Published:** 2009

**Authors:** Md Quamar Azam, AA Iraqi, MKA Sherwani, M Abbas, Afzal Alam, Amir Bin Sabir, Naiyer Asif

**Affiliations:** Department of Orthopaedic Surgery, J. N. Medical College, Aligarh Muslim University, Aligarh, Uttar Pradesh, India

**Keywords:** Delayed fixation, femoral neck fracture, pediatric fractures

## Abstract

**Background:**

Time from injury to fixation of femoral neck fractures has been postulated as a vital determinant for rate of complications; however, no prospective study is available in the English literature. Delay, unfortunately, is inevitable in developing countries. The aim of the present study is to retrospectively review the outcome after delayed fixation of displaced type II and III femoral neck fractures in children.

**Materials and Methods:**

Using a standard assessment chart, we retrospectively reviewed medical records of all pediatric patients having femoral neck fractures presenting to our institution from June 1999 to May 2006. Inclusion criteria were children between 5 and 15 years of age sustaining displaced Delbet type II and III femoral neck fractures having a complete follow-up of at least 2 years. Patients with known metabolic disease, poliomyelitis or cerebral palsy, were excluded from the study. After application of inclusion and exclusion criteria, 22 patients having 22 fractures (13 type II and 9 type III) were studied. Surgery could be performed after a mean delay of 11.22 days (ranging from 2 to 21 days). Closed reduction was achieved in 14 cases and 8 cases required open reduction through anterolateral approach.

**Result:**

Osteonecrosis was noted in eight patients (36.37%) who included two of nine patients (22.22%) operated in the first week, three of eight patients (37.51%) operated in the second week, and three of five patients (60%) operated in the third week of injury. Nonunion was seen in four (18.18%) cases, and two of them were associated with failure of implants. One was treated by valgus osteotomy and the other by Meyer's procedure. Fractures united in both children but the latter developed avascular necrosis. Functional results, as assessed using Ratliff's criteria, were good in 14 (63.63%), fair in 2 (9%), and poor in 6 (27.27%) patients.

**Conclusion:**

Delay in fixation, type of fracture, and ability to achieve and maintain reduction are factors primarily responsible for the outcome. We also found that delay after the first week further adversely affects the outcome.

## INTRODUCTION

Femoral neck fracture in children is an exceedingly uncommon injury,^1^ sinister in nature,^2^ and beset with frequent complications^3^. The proximity of femoral epiphysis, vulnerable blood supply,^4^,^5^ and extreme degree of forces involved are primary reasons for high incidence of complications like avascular necrosis (AVN), coxa vara, limb length discrepancies, and nonunion. Type of fracture, displacement, age of child, and ability to achieve and maintain reduction are accepted factors directly responsible for the outcome. The aim of the present study is to evaluate the outcome in type II and III, displaced femoral neck fractures in children who underwent surgery after at least 48 h of delay.

## MATERIALS AND METHODS

Using a standard assessment chart, we retrospectively reviewed medical records of all pediatric patients having femoral neck fractures presenting to our institution from June 1999 to May 2006. Medical records were reviewed to determine gender, age, mechanism, time and date of injury, and time and date of surgery performed. Initial radiographs were analyzed for fracture classification. Inclusion criteria were children between 5 and 15 years of age sustaining displaced Delbet type II and III femoral neck fractures and a follow-up of at least 2 years. Patients with known metabolic disease, poliomyelitis or cerebral palsy, were excluded from the study. Our initial search recorded 48 displaced femoral neck fractures, which included 2 type I, 25 type II, 14 type III, and 7 type IV. After application of inclusion and exclusion criteria, 22 patients having 22 fractures (13 type II and 9 type III) were identified [Table 1].

**Table 1:** Details of patients with clinical and radiographic follow up

Cases	Age/Sex	Mechanism of injury	Fracture type	Delay (days)	Treatment	Follow-up (years)	Complications
1	12/M	Struck by auto	II	11	OR and IF	6	ON
2	9/M	Fall from roof	II	6	OR and IF	8	-
3	7/F	Fall from wall	III	4	CR and IF	7	-
4	10/F	Car vs. pedestrian	II	17	OR and IF	3	ON, NU
5	8.5/M	Fall from tree	III	21	CR and IF	9	-
6	13 /M	While playing	III	3	OR and IF	5	NU (united after osteotomy)
7	5/M	Bicycle accident	II	10	CR and IF	4	-
8	15/F	Bike vs. bus	III	8	OR and IF	2 1/2	ON
9	6.5/M	Fall from rickshaw	II	9	OR and IF	9	-
10	8/F	Fall from wall	II	17	CR and IF	4	ON
11	7/F	While playing	III	6	CR and IF	3 1/2	-
12	9/M	Motor bike pillion	II	2	CR and IF	8	ON, NU
13	9.5/M	Bicycle accident	III	5	CR and IF	6	-
14	8.5/M	Thrown out of auto	III	8	CR and IF	4	-
15	13/M	While playing	II	10	OR and IF	5	-
16	9/F	Bike vs. auto	II	12	CR and IF	6	ON
17	8/M	Fall from wall	II	7	CR and IF	8 1/2	-
18	7/F	Fall from rickshaw	II	11	CR and IF	7 1/2	-
19	6.5/F	Thrown out of auto	III	7	CR and IF	9	-
20	10/M	Car vs. pedestrian	II	18	OR and IF	5 1/2	-
21	9.5/M	Fall from tree	III	19	CR and IF	2	ON, NU (united after Meyer's procedure)
22	8/M	Motor bike pillion	II	5	CR and IF	7	ON

CR= closed reduction, OR= open reduction, IF= internal fixation, ON= osteonecrosis, NU= nonunion.

The mechanisms of injuries were road accidents in nine (40.90%), fall from height (roof and tree) in six (27.27 %), fall from bicycle/rickshaw in four (18.18%), and while playing in three (13.63%) children. Surgery was performed after a mean delay of 11.22 days (ranging from 2 to 21 days). Nine patients were operated in the first week; eight were operated in the second week and the remaining five in the third week. The cause of the delay included late presentation in 13 cases (59%), associated injuries in 5 (22.72%), and lack of facilities in the emergency operation theatre in 4 (18.18%). All operations were performed by three senior most authors. Closed reduction could be achieved in 14 cases under fluoroscopic control. In the remaining eight cases, open reduction was performed through an anterolateral approach. Hip spica was applied for 6 weeks in 17 cases where the age was less than 10 years. Traction was not applied in any case after surgery.

Bedside exercises like quadriceps drill and range of motion exercises were started from the second day. Dressing was changed on 4^th^ postoperative day, and patients were discharged between 12^th^ and 14^th^ postoperative days after stitch removal. Nonweight bearing crutch walking was started from the third postoperative day (where spica was not given) and full weight bearing allowed after achieving union. Patients were followed every 6th week till union was achieved, then every 3 months till 2 years and then every 6 months. Duration of the follow-up ranged from 2 to 9 years (mean 5.98 years).

Postoperative radiographs were closely analyzed to determine accuracy of reduction. Reduction quality was determined using the following scale^6^: excellent = less than 2-mm step off and no angulation; good = less than 4-mm step off and less than 5° angulation; fair = greater than 4-mm step off and less than 10° of angulation; poor = greater than 5-mm step off and greater than 10° of angulation. Of the 22 patients, 15 had excellent reduction, 5 good and 2 fair reductions.

In the follow-up, a detailed history to include pain and difficulty in performing any specific activity was recorded. An examination was performed to reveal any tenderness, limitation in range of motion, and leg length discrepancies. Radiographs were reviewed to determine union (evidenced by bridging trabeculae across the fracture site), osteonecrosis, and angular deformity. Coxa vara and valga were defined^7^ as neck shaft angles of ≤130° and ≥150°, respectively.

## RESULTS

**Figure 1:**
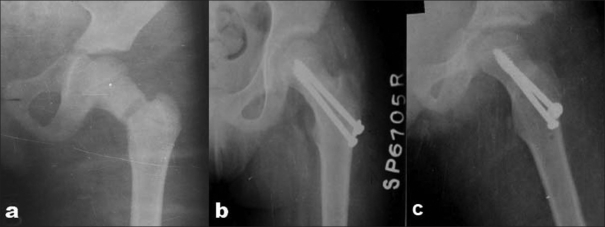
(a) X-ray hip joint (anteroposterior view) showing displaced femoral neck fracture (type II) in a 9-year-old boy, presented after 4 days of injury. (b,c) Follow-up radiographs (anteroposterior and lateral view) after 30 months showing union without any signs of AVN

**Figure 2:**
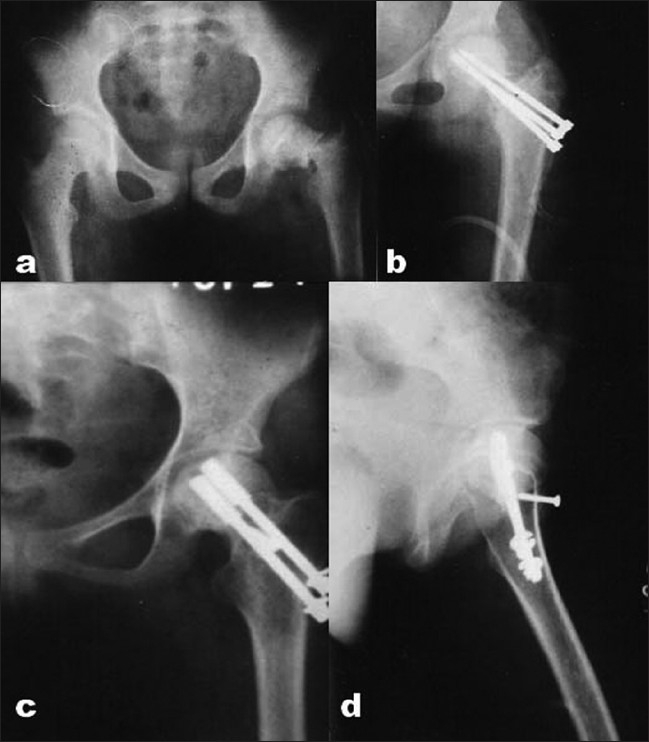
(a) X-ray pelvis with both hip joints (anteroposterior view) showing displaced femoral neck fracture in a male of 9½ years presented after 7 days. (b) An anteroposterior view showing nonunion with failed implants. (c,d) Follow-up radiographs (anteroposterior view and lateral view) at 28 months showing union after Meyer's procedure. The femoral head later on went into AVN

**Figure 3:**
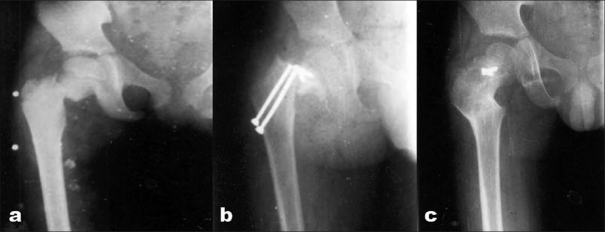
(a) Preoperative radiograph of right hip joint (anteroposterior view) of a 13-year-old boy showing a displaced femoral neck fracture who presented after 5 days. (b) X-ray hip joint (anteroposterior view) of the same patient showing breakage of implant at 18 weeks. (c) A radiograph hip joint (anteroposterior view) of showing union after implant (distal part) removal and valgus osteotomy

Union was achieved in 16 patients [Figure 1]. Nonunion was seen in four (18.18%) patients; this included two patients (cases 4 and 6) of open reduction and two cases of closed reduction (cases 12 and 21). Two patients (cases 4 and 12) had type II fracture and other two patients (cases 6 and 21) had type III. Two of them had nonunion with implant failure. One (case 21) was treated by Meyer's procedure [Figure 2] and the other (case 6) by valgus osteotomy [Figure 3]. Fracture united in both of them; however, the first child developed osteonecrosis of the femoral head. Despite revision surgery (osteotomy), union could not be achieved in remaining two children (cases 4 and 12). Both of them were type II; closed reduction was successful in one while the other required open reduction. Both of them continued walking with limp till the last follow up.

AVN was seen in eight patients (36.36%); this included 6 (46.15%) of the 13 having type II fractures and 2 (22.22%) of the 9 having type III fractures. Closed analysis of these patients revealed that five developed AVN despite excellent reduction and remaining three developed AVN where reduction was subsequently lost in the follow-up. AVN developed in 3 (37.5%) of the 8 patients where open reduction was performed, while 5 (35.71%) of the 14 patients in the closed reduction group developed AVN. Eight patients who developed AVN included two (both type II) of the nine patients (22.22%) operated in the first week, three of the eight cases (46.15%) operated in the second week, and another three of five children (60%) operated in the third week, after injury. Results were assessed using Ratliff's criteria [Table 2] as this is being widely accepted. Fourteen (63.63%) patients showed good results, while two (9%) had fair and six (27.27%) poor results.

**Table 2:** Ratliff's criteria for functional assessment of the result of treatment for fracture of the hip

	Good	Fair	Poor
Pain	None or “ignore”	Occasional	Disabling
Movement	Full or terminal restriction	Greater than 50%	Less than 50%
Activity	Normal or avoids game	Normal or avoids game	Restricted
Radiographic findings	Normal or some deformity of the neck	Severe deformity of the femoral neck	Severe avascular necrosis, degenerative arthritis, arthrodesis

## DISCUSSION

Neglected femoral neck fracture is commonly seen in developing countries as patients have tendency to go to bone setters who mismanage.^8^–^10^ The various reasons for such delay are ignorance, financial constraints, and firm belief of patients in village bone setters. A large waiting list at the referral center and lack of adequate emergency facilities for dealing with such fractures adds to further delays in surgery. A high incidence of complications is reported^11^ due to kinking of vessels (rather than tear) of the proximal epiphysis; therefore, early reduction is desirable for a better outcome. Besides this, we believe that when fixation is delayed, it requires added manipulation to achieve closed reduction or more soft tissue dissection, and this results in further vascular insult, which adversely affects the outcome. This possibly explains why our five patients developed AVN despite excellent reduction. Although the time from injury to fracture reduction has been postulated to be a vital factor in determining whether a pediatric patient will develop AVN, no prospective studies are available in the English language literature, which has evaluated impact of reduction and timing on the development of AVN.^12^ Shrader *et al*^12^ in their retrospective study found that risk of AVN increases with increased time to reduction. None of their 15 patients who were operated within 24 h developed AVN; in contrast, 2 of the 5 cases operated after 48 h had AVN. Further analysis of their two cases showed that one of the patients had type IA and the other type II fracture, and the reduction achieved in them was either poor or fair and implants used were of older generation (Canakis and Knowles pin). Delay in surgery thus cannot be totally blamed for the outcome.

The rate of AVN was 36.37% in our series, which is slightly better than that reported earlier.^13^,^14^ This is attributed to the better imaging facility, improved implants, and exclusion of Delbet type I fracture. However, our results are inferior to the recent reports in the literature^12^,^15^,^16^ for two reasons: first, the fixation was done after a mean delay of 11.22 days and second, only displaced type II and type III fractures are included in the present series. AVN developed in three cases where reduction was subsequently lost. However, in another five patients, AVN developed despite excellent reduction, of which two were operated in the second week and three in the third week. Three of them were type II while two were type III. Three underwent closed reduction and two open reductions. We attribute delayed fixation as a contributing factor after considering other variables. Rate of AVN was not statistically significant in the open reduction group (37.5%) when compared to the closed reduction group (35.71%). Dhammi *et al*^17^ noted that average fracture union time in the open reduction group (10.2 weeks) was better than that in the closed reduction group (12.6 weeks). However, they concluded that there was no statistically significant difference in the surgical outcome between the two groups. Predicting AVN remains controversial as there are multiple mechanisms that have the potential to cause a necrotic event. Insult to vascular supply (during injury/manipulation), displacement, age, and treatment method are all strong independent predicators. Moon *et al*^18^ in a structured meta-analysis of 360 cases concluded that the fracture type and age are only two statistically significant variables. It is widely recognized that percentage of good result is inversely proportional to the degree of displacement of the fracture.^19^–^21^ However, AVN does also occur in undisplaced or even incomplete fractures.^1^,^2^ Pforringer and Rosenmeyer^22^ and Kay *et al*^23^ observed that adolescents are at a greater risk of AVN because they still possess tenuous vascular supply without the potential ability to revascularize and remodel the femoral head.

Lam's^20^ series of 75 patients included 60 fresh cases (who arrived hospital within 5 days) and 15 late (reached between 7 days and 8 months). AVN recorded by him was 18% in type II and 22% in type III fractures. He concluded that displaced trans-cervical and cervico-trochanteric fractures remain an unsolved problem. He opined that if closed reduction succeeds, hip spica is adequate in younger patients. However, in older children, internal fixation reinforced by spica is preferable.

Canale and Bourland^13^ reported that the incidence is especially high in Delbet type I (100%) and type II (50%) and lower in type III (27%) and type IV (14%) fractures. They found that 96% of fractures in their series which developed AVN were displaced. Ratliff^19^ reported that 71% of displaced fractures developed complication. Using Ratliff's criteria for functional assessment, good result was obtained in 63.63% of our cases.

Surgical decompression of the capsule remains a subject of debate. Cheng and Tang^15^ and Swiontkowski and Winquist^11^ achieved better result with early evacuation of hematoma, whereas Flynn^16^ concluded that incidence of necrosis is low if fracture is promptly reduced and stabilized even without decompression.

Coxa vara as a complication to fractures of the neck of femur in children is reported to be between 14 and 32%,^13^,^19^,^20^ and that of nonunion as 6.5%,^13^ 27%^20^ 33%^19^ and premature closure between 20%^19^,^20^ and 62%.^13^ In our series, coxa vara was observed in 18.18% of cases. Various authors are unanimous in concluding that the incidence of these complications increases if treated conservatively. With the increase in the trend of internal fixation in pediatric femoral neck fractures using second-generation implants, rates of these complications are significantly reduced. Flynn^16^ had coxa vara in none of his patients whereas nonunion in one (6%).

Limitations of this study are that sample size in each group is small, so statistical analysis is not possible. It is an observational study and scientific analysis needs further evaluation.

## CONCLUSION

In conclusion, when surgery was delayed for more than 48 h, complications were higher when compared to the recent series in the literature where the fracture was fixed within 24 h. We further observed that the rate of AVN progressively increases from the first to the third week. A multicenter prospective study of cases where surgery is delayed due to unavoidable circumstances would certainly provide a better answer.
